# Effects of Exogenous Melatonin on Methyl Viologen-Mediated Oxidative Stress in Apple Leaf

**DOI:** 10.3390/ijms19010316

**Published:** 2018-01-21

**Authors:** Zhiwei Wei, Tengteng Gao, Bowen Liang, Qi Zhao, Fengwang Ma, Chao Li

**Affiliations:** State Key Laboratory of Crop Stress Biology in Arid Areas, College of Horticulture, Northwest A&F University, Yangling 712100, Shaanxi, China; weizhiwei@nwsuaf.edu.cn (Z.W.); gaotengteng@nwsuaf.edu.cn (T.G.); liangbowen@nwsuaf.edu.cn (B.L.); zhaoqi93@nwsuaf.edu.cn (Q.Z.)

**Keywords:** *Malus*, melatonin, reactive oxygen species, oxidative stress, methyl viologen

## Abstract

Oxidative stress is a major source of damage of plants exposed to adverse environments. We examined the effect of exogenous melatonin (MT) in limiting of oxidative stress caused by methyl viologen (MV; paraquatin) in apple leaves (*Malus domestica* Borkh.). When detached leaves were pre-treated with melatonin, their level of stress tolerance increased. Under MV treatment, melatonin effectively alleviated the decrease in chlorophyll concentrations and maximum potential Photosystem II efficiency while also mitigating membrane damage and lipid peroxidation when compared with control leaves that were sprayed only with water prior to the stress experiment. The melatonin-treated leaves also showed higher activities and transcripts of antioxidant enzymes superoxide dismutase, peroxidase, and catalase. In addition, the expression of genes for those enzymes was upregulated. Melatonin-synthesis genes *MdTDC1*, *MdT5H4*, *MdAANAT2*, and *MdASMT1* were also upregulated under oxidative stress in leaves but that expression was suppressed in response to 1 mM melatonin pretreatment during the MV treatments. Therefore, we conclude that exogenous melatonin mitigates the detrimental effects of oxidative stress, perhaps by slowing the decline in chlorophyll concentrations, moderating membrane damage and lipid peroxidation, increasing the activities of antioxidant enzymes, and changing the expression of genes for melatonin synthesis.

## 1. Introduction

Environmental stress is becoming one of the most severe agricultural problems affecting plant growth and crop yield [1]. Being sessile, plants are generally exposed to recurrent cycles of injury from a variety of biotic and abiotic stresses. Methyl viologen (MV; also called paraquat) can inhibit photosynthesis [2]. This non-selective redox herbicide is widely applied around the world to prevent the growth of broadleaf weeds and grasses, and is commonly used in studies of the relationship between photosynthesis and oxidative stress [3].

Methyl viologen is a strong auto-oxidable electron acceptor in Photosystem I (PSI); its presence in the chloroplasts of light-exposed plants has several important consequences [4]. It catalyzes the generation of superoxide radicals by accepting electrons from PSI, thus inhibiting the reduction of ferredoxin, and then transfers the electrons to oxygen-forming superoxide radicals that, via a disproportionation reaction, are converted to H_2_O_2_, leading to oxidative stress [4,5]. Exposure of leaves to MV produces rapid chlorophyll loss and evident necrosis of the tissues. Methyl viologen can also cause lipid peroxidation, protein denaturation, inhibition of photosynthesis, and electrolyte leakage due to the loss of membrane integrity [6,7]. Malondialdehyde (MDA) can serve as an indicator of lipid peroxidation damage caused by MV stress [8]. In the presence of MV, antioxidant enzymes are usually induced in leaves due to the generation of superoxide [9].

Melatonin (*N*-acetyl-5-methoxytryptamine), a tryptophan-derived natural product, is a pleiotropic molecule with numerous cellular and physiological actions in almost all living organisms, including animals and plants [10]. In humans and other animals, this remarkable molecule not only signals the time of day or year, but also promotes immunomodulatory and cytoprotective properties [11]. Since melatonin was first reported in higher plants [12,13], its use in numerous studies has led to an accumulation of information about its possible physiological functions. For example, melatonin regulates multiple developmental processes and defenses against stresses such as extreme temperatures, chemical pollutants, alkaline conditions, salinity, drought, and fungal pathogens [8,14,15,16,17,18]. As an antioxidative molecule, its also has a significant role in scavenging free radicals, especially reactive oxygen and nitrogen species [11]. 

Melatonin is sequentially synthesized from tryptophan in four steps: decarboxylation by tryptophan decarboxylase (TDC), hydroxylation of tryptamine by tryptamine 5-hydroxylase (T5H) to serotonin, *N*-acetylation by a serotonin *N*-acetyltransferase (SNAT) (also called arylakylamine *N*-acetyltransferase (AANAT)), and, finally, *O*-methylation to melatonin by *N*-acetylserotonin *O*-methyltransferase (ASMT), formerly known as hydroxymethyl *O*-methyltransferase [19,20,21,22,23,24]. Genes for the first two enzymes were initially cloned and characterized in rice (*Oryza sativa*) [21,22]. Furthermore, AANAT was first identified in the unicellular green alga *Chlamydomonas reinhardtii* [23] and ASMT was first cloned and characterized in rice by Kang et al. [20].

A few studies have focused on the roles of exogenous melatonin in regulating plant defenses against oxidative stress. Pretreatment with melatonin can alleviate photo-oxidative damage by enhancing oxidative stress-induced autophagy [25]. Exogenous melatonin can also protect against MV-mediated oxidative stress in cucumber (*Cucumis sativus*) and is involved in redox signaling [26]. This suggests that exogenous melatonin induces both local and systemic tolerances against oxidative stress. However, questions remain about the physiological and biochemical changes during MV-induced stress when leaves are pre-treated with melatonin, as well as about how the expression of melatonin synthesis genes and antioxidant enzymes change in those leaves.

Here, we examined the role of melatonin in detached apple leaves upon MV-mediated oxidative stress. We also explored the effect of different melatonin concentrations and monitored changes in the activities of several antioxidant enzymes, levels of H_2_O_2_, and the expression of genes involved in melatonin synthesis. Our main focus in this report was on physiological and biochemical changes in the plants and alterations in gene expression when leaves were pre-treated with melatonin prior to MV-induced stress.

## 2. Results

### 2.1. The Effect of Melatonin on Phenotypes of Apple Leaves under MV Stress

**Figure 1:**
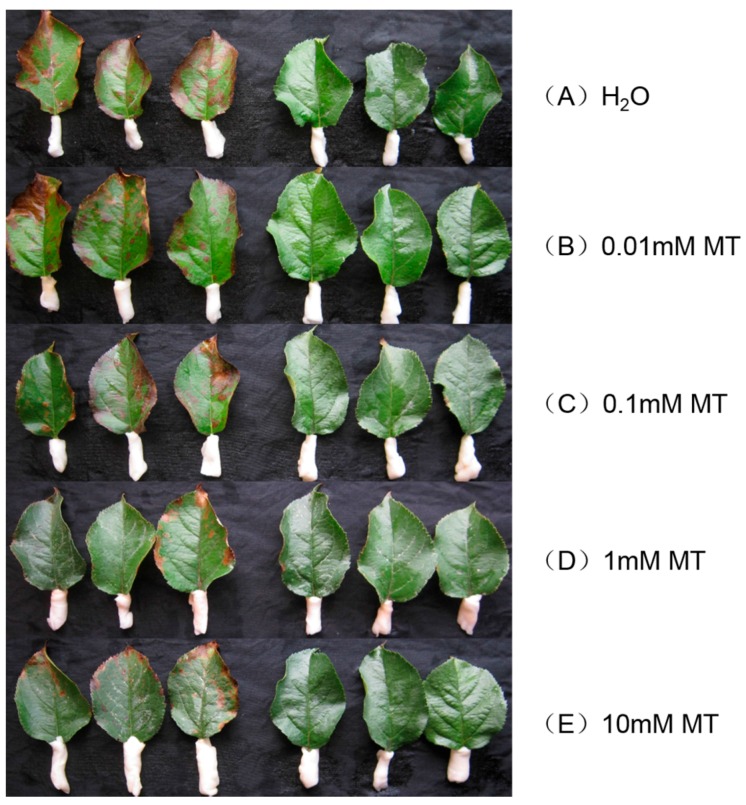
Effect of exogenous melatonin (MT) on apple leaf phenotype at 48 h after MV treatment. Fully mature leaves were collected from plants pre-treated with water only (**A**); or with MT concentrations of 0.01 mM (**B**); 0.1 mM (**C**); 1 mM (**D**); or 10 mM (**E**).

**Figure 2:**
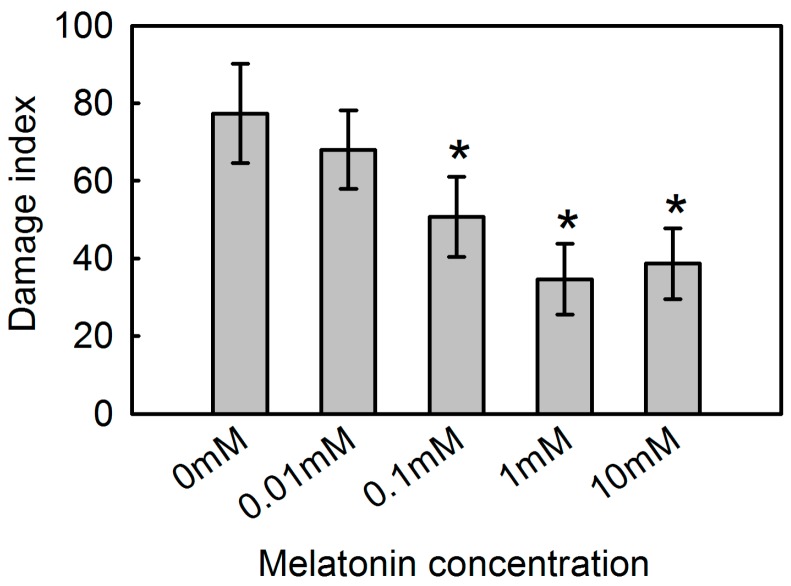
Effect of exogenous melatonin on damage index at 48 h after MV treatment for apple leaves pre-treated with 0 mM, 0.01 mM, 0.1 mM, 1 mM, or 10 mM melatonin. Data represent means ± SD of 15 replicate samples. *, significant difference (*p* < 0.05) between plants pre-treated with and without melatonin, based on Tukey’s multiple range test.

To analyze the protective effect of melatonin against MV-mediated oxidative stress in apple, we pre-treated detached leaves for 12 h with different concentrations of melatonin (0 mM, 0.01 mM, 0.1 mM, 1 mM, or 10 mM). After 48 h of exposure to MV, samples from plants receiving no melatonin showed rapid chlorophyll loss and evident necrosis (Figure 1). However, the addition of 0.01 mM to 10 mM melatonin alleviated such damage to varying degrees, as defined by the extent and spread of lesions. Compared with leaves that were not pre-treated, those in the 1 mM melatonin sub-group showed the greatest decline (42.6%) in the calculated damage index while damage was decreased by 38.6% for those in the 10 mM sub-group (Figure 2).

### 2.2. The Effect of Melatonin on Physiological State of Apple Leaves under MV Stress

**Figure 3:**
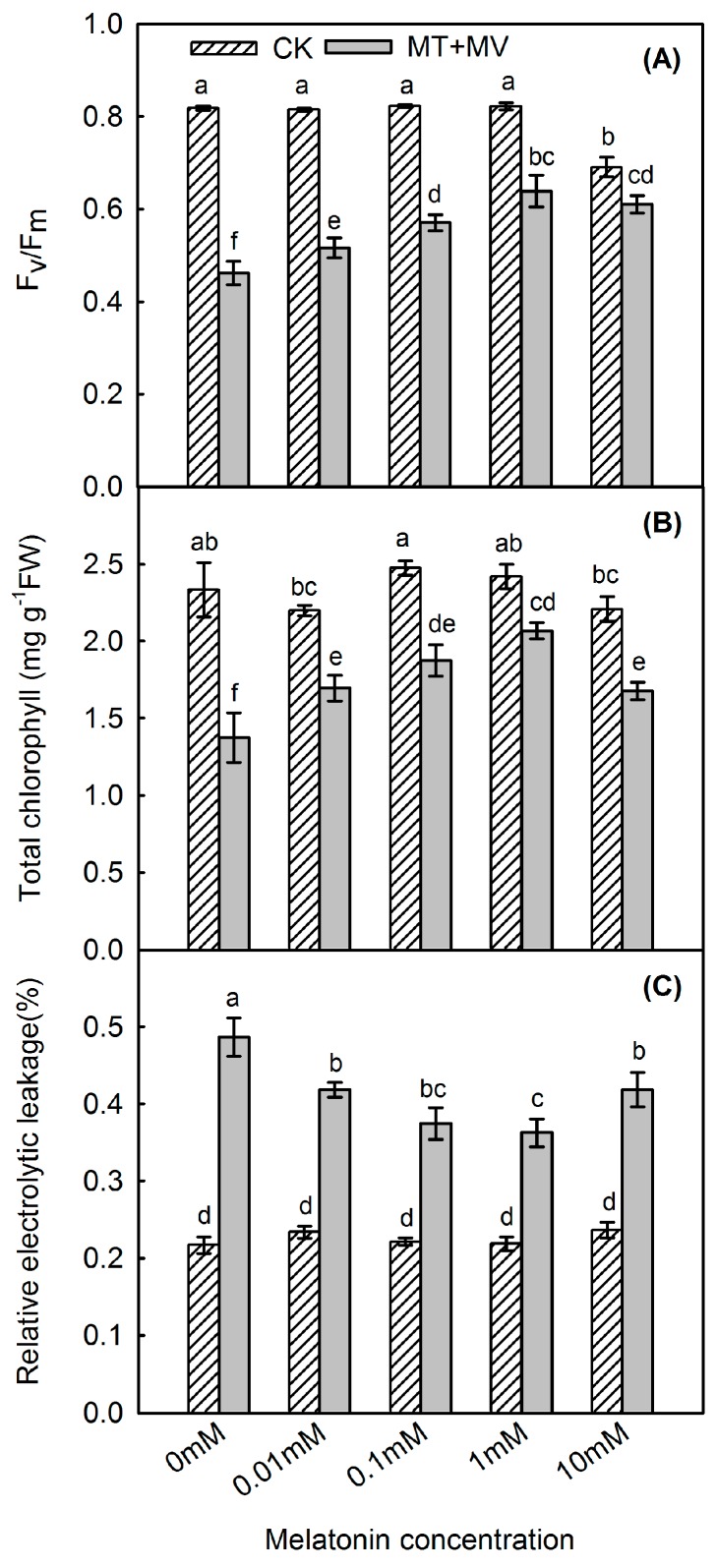
Effect of exogenous melatonin on *F*_v_/*F*_m_ (**A**); total chlorophyll concentration (**B**); and leaf relative electrolyte leakage (**C**) at 48 h after MV treatment for apple leaves pre-treated with 0 mM, 0.01 mM, 0.1 mM, 1 mM, or 10 mM melatonin. Values are means of 3 replicates ± SD. Different letters indicate means are significantly different (*p* < 0.05), based on ANOVA and Tukey’s test.

We examined the role of exogenous melatonin in protecting against MV action by measuring maximum potential Photosystem II (PSII) efficiency (*F*_v_/*F*_m_), total chlorophyll, and leaf relative electrolyte leakage (REL) in apple leaves. Treatment with MV diminished *F*_v_/*F*_m_ by 43.5%, but that decrease was tempered by the melatonin (Figure 3A), especially at the concentration of 1 mM. Chlorophyll metabolism was also influenced by MV. For example, at 48 h post-treatment, the total amount of chlorophyll was 58.9% of that measured in the control in MV treatment, whereas it was 85.4% in the 1 mM melatonin pre-treated group (Figure 3B). Relative electrolyte leakage can be used to reflect the extent of damage to leaf membranes. At Hour 48 of MV treatment, REL was significantly increased, by 124.4%, when compared with the control. However, that rise was alleviated by melatonin, especially at 1 mM, a level that tempered this increase by 67.3% when compared with the control (Figure 3C).

**Figure 4:**
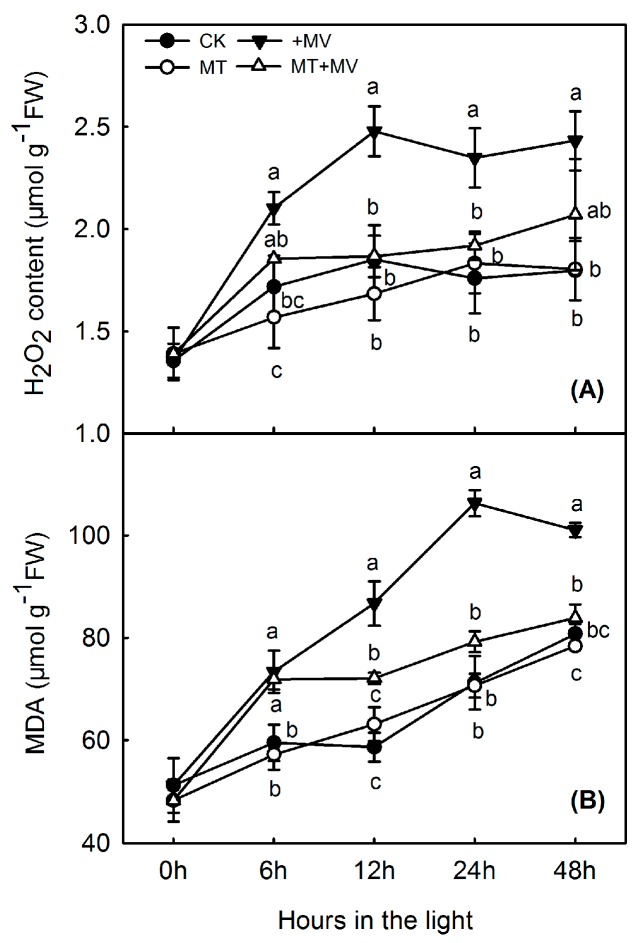
Effect of 1 mM melatonin on levels of H_2_O_2_ (**A**) and MDA (**B**) in apple leaves during MV treatment. Values are means of 3 replicates ± SD. Different letters indicate means are significantly different (*p* < 0.05), based on ANOVA and Tukey’s test.

Based on the results above, we selected 1 mM melatonin for further study. H_2_O_2_ concentration rose slightly in the control and this rise was diminished in the first 12 h by adding melatonin. Compared with the controls, MV exposure increased leaf H_2_O_2_ concentrations under all experimental conditions (Figure 4A), with levels rising by 34.1% after 12 h and remaining high thereafter. Under oxidative stress, however, exogenous melatonin significantly suppressed H_2_O_2_ production. Relative to activity in the control leaves, oxidative stress increased leaf MDA during all treatments, similar to the trend noted with H_2_O_2_ (Figure 4B). MDA in the control rose steadily and significantly over the 48 h of light, but this rise was not affected by adding melatonin. After 12 h of oxidative stress, MDA levels increased by 47.9% when compared with the control, but that response was significantly suppressed in leaves that had been pre-treated with 1 mM melatonin.

### 2.3. The Effect of Melatonin on the Activity of Antioxidant Enzymes in Apple Leaves under MV Stress

**Figure 5:**
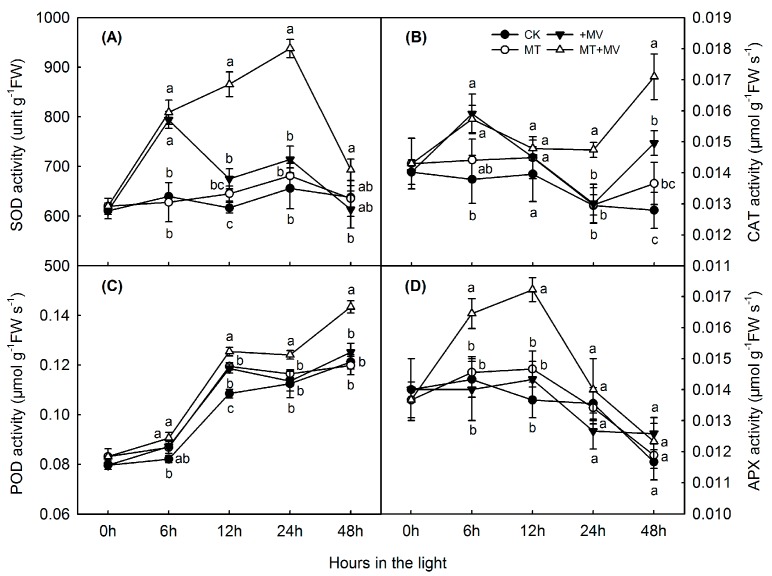
Effect of 1 mM melatonin on activity of main antioxidative enzymes in apple leaves during MV treatment: SOD (**A**); CAT (**B**); POD (**C**); and APX (**D**). Values are means of 3 replicates ± SD. Different letters indicate means are significantly different (*p* < 0.05), based on ANOVA and Tukey’s test.

Figure 5 presents the results from our tests of the effects of MV and exogenous melatonin on antioxidant activity, in particular the enzymes involved in scavenging reactive oxygen species (ROS). As the period of oxidative stress was prolonged, activities of superoxide dismutase (SOD), catalase (CAT), and ascorbate peroxidase (APX) in the leaves first increased and then decreased gradually (Figure 5A,B,D), while peroxidase (POD) activity continued to slowly increase over time (Figure 5C). SOD and CAT remained steady in control over the 48 h of light and the levels were not affected by adding melatonin. POD rose steadily in the control, with slight increase with melatonin addition at 12 h. APX remained steady in the control for 12 h and then fell and the addition of melatonin had no effect. Pretreatment with melatonin led to significantly higher activities for SOD (12 and 24 h), CAT (24 and 48 h), APX (6 and 12 h), and POD (12, 24, and 48 h) during the MV treatments.

### 2.4. The Effect of Melatonin on the Expression of Related Genes in Apple Leaves under MV Stress

**Figure 6:**
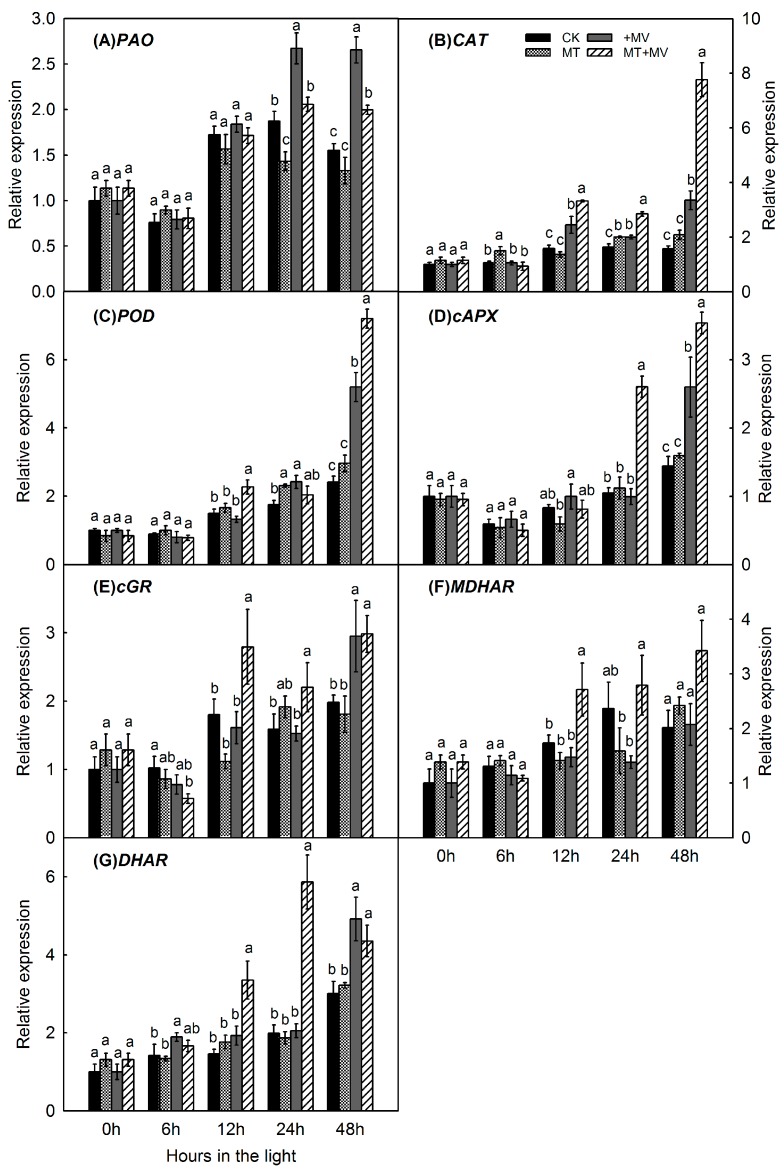
Effect of 1 mM melatonin on relative expression of *pheide A oxygenase*(*PAO*) (**A**); *CAT* (**B**); *POD* (**C**); *cAPX* (**D**); *cGR* (**E**); *MDHAR* (**F**); and *DHAR* (**G**) in apple leaves during MV treatment. Total RNA was isolated from samples taken at different time points, converted to cDNA, and subjected to real-time RT-PCR. Expression levels were calculated relative to expression of *Malus EF-1α* mRNA. Values are means of 3 replicates ± SD. Different letters indicate means are significantly different (*p* < 0.05), based on ANOVA and Tukey’s test.

Pheide A oxygenase (PAO), a nuclear-encoded enzyme, is induced by a key chlorophyll degradation gene [27]. Compared with transcripts in the control leaves, relative expression of *PAO* was upregulated by MV stress. However, pretreatment with 1 mM melatonin markedly inhibited the expression of *PAO* genes after 24 and 48 h of exposure to MV (Figure 6A). For better understanding of these changes in levels of antioxidant enzymes, we monitored the expression of related genes. The antioxidant gene expression tended to increase in the control over the 48 h of light, with no clear effect by added melatonin. Compared with control leaves, *CAT* and *POD* expression rose over time, with exogenous melatonin leading to respective increases of 4.9-fold and 3.0-fold after 48 h of MV treatment (Figure 6B,C). As the stress period was prolonged, expression of *cAPX* and *cGR* first decreased and then increased gradually (Figure 6D,E). Exogenous melatonin increased their expression by 2.5-fold and 1.4-fold, respectively, after 24 h. During the entire stress experiment, *MDHAR* and *DHAR* expression continued to increase, and melatonin pretreatment was associated with significant increases after 12 h (*MDHAR*) and 48 h (*DHAR*) (Figure 6F,G).

**Figure 7:**
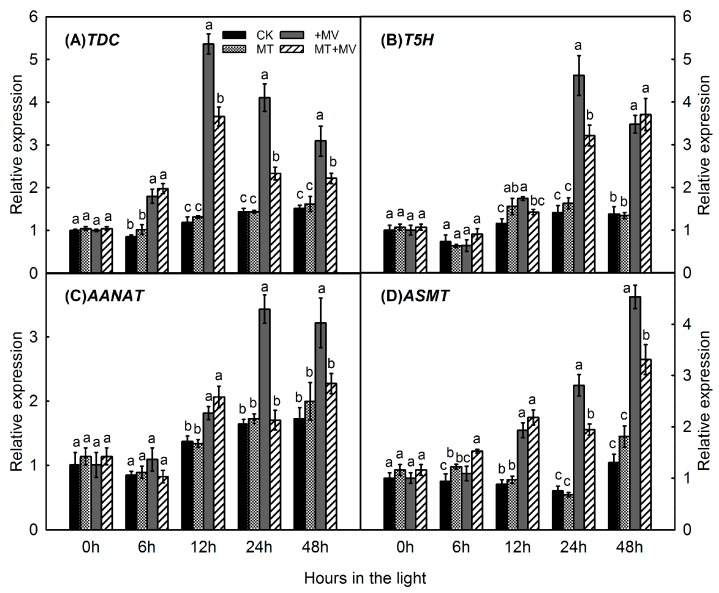
Effect of 1 mM melatonin on relative expression of melatonin synthesis genes—*MdTDC1* (**A**); *MdT5H4* (**B**); *MdAANAT2* (**C**); and *MdASMT1* (**D**)—in apple leaves during MV treatment. Total RNA was isolated from samples taken at different time points, converted to cDNA, and subjected to qRT-PCR. Expression levels were calculated relative to expression of *Malus EF-1α* mRNA. Values are means of 3 replicates ± SD. Different letters indicate means are significantly different (*p* < 0.05), based on ANOVA and Tukey’s test.

Because melatonin in plants is synthesized from tryptophan sequentially by four enzymes, we investigated the expression of genes involved in its production. In the absence of MV, expression of *MdTDC1* (Figure 7A), *MdT5H4* (Figure 7B), *MdAANAT2* (Figure 7C), and *MdASMT1* (Figure 7D) did not differ significantly between pre-treated and control leaves. The gene expression involved in the melatonin synthesis pathway did not change greatly in the control and was not affected by melatonin addition. When MV stress was induced, all of these genes were significantly upregulated. However, pretreatment with 1 mM melatonin significantly suppressed the expression of *MdTDC1* (12, 24, and 48 h), *MdT5H4* (12 and 24 h), *MdAANAT2* (24 and 48 h), and *MdASMT1* (24 and 48 h) during the MV treatments.

## 3. Discussion

As a ubiquitous compound, melatonin has an important role as an antioxidant that protects plants from the damaging effects of ROS [28]. Melatonin alleviates the effects of oxidation induced by environmental stresses [26,29]. Our results demonstrated that, in a dose-dependent manner, exogenous melatonin clearly plays a role in protecting apple leaves exposed to MV, reducing the degree of lesions and slowing their rate of expansion. Similarly, in *Lycopersicon esculentum*, exogenous melatonin (dose-dependent) increased plant tolerance to sodic alkaline stress [30]. Methyl viologen is a non-selective herbicide frequently used by plant researchers to investigate relationships among oxidative tolerance, cross-tolerance responses, and the antioxidant system [31]. In light-exposed plants, MV can transfer electrons from photosystem I to molecular oxygen [7]. In this study, focused on the roles of exogenous melatonin in regulating plant defenses against oxidative stress. 

To examine the MV-induced photosynthetic apparatus damage in plant, we monitored the value of *F*_v_/*F*_m_. Maximum potential PS II efficiency, calculated as the ratio of variable fluorescence to maximum fluorescence, can be used to reflect the extent of damage to the photosynthetic apparatus under stress conditions [32]. We found that exogenous melatonin helped maintain *F*_v_/*F*_m_ values during the subsequent oxidative stress period, and pre-treated leaves contained more total chlorophyll than the control leaves. The chlorophyll-preserving influence of melatonin has also been confirmed with apple leaves during salinity stress or senescence [16,27], in a photosynthetic green macroalga (*Ulva* sp.) exposed to toxic conditions [33], and in cucumber seedlings subjected to photo-oxidative stress [26]. We also found that exogenous melatonin can preserve the content of chlorophyll by downregulating the expression of *PAO*, a chlorophyll degradation gene located in the envelope membrane of gerontoplasts [27]. This preservation of chlorophyll status via downregulation has also been reported for another degradation gene, *chlorophyllase* (*CLH1*) [27,34]. Therefore, all of these data for chlorophyll and *PAO* expression clearly demonstrate that the addition of melatonin slows chlorophyll degradation in MV-stressed leaves. 

Melatonin is an amphipathic molecule and can easily diffuse through cell membranes and enter subcellular compartments. Melatonin can reduce lipid peroxidation and maintain membrane stability in plants [10,11,16]. Electrolyte leakage is a good indicator of membrane permeability or loss of membrane integrity under stress conditions [16]. To quantify the oxidative damage caused by MV and the protective role of melatonin, we calculated REL in apple leaves and found that values were much higher in the 0 mM sub-group than other sub-groups after MV treatment, indicating that pretreated leaves had greater membrane stability. The melatonin also exhibited a dose-dependent relationship, with the most effective concentration being 1 mM. Higher or lower concentrations possibly attenuated that response. As previously reported in drought-stressed apple, REL was significantly lower in leaves from melatonin-pre-treated plants when compared with the control [29].

Melatonin works as a growth promoter and antioxidant molecule under stress conditions [30], and is widely used as a natural substance in agricultural additives [35]. Reactive oxygen species are pivotal in regulating numerous biological processes such as growth, development, and responses to biotic and abiotic stresses. Oxidative stress arises from an imbalance between ROS production and the detoxification of their reactive intermediates, which is of vital importance if plants are to maintain intracellular ROS pools at low levels [36]. Various mechanisms control temporal and spatial coordination between ROS and other signals that are activated in separate parts of the plant at different times [28]. As the most important ROS, H_2_O_2_ participates in a series of processes for plant development, stress responses, and programmed cell death [29]. We noted here that H_2_O_2_ concentrations were significantly increased in the control leaves after MV treatment when compared with those pre-treated with a relatively low level in melatonin. This exogenous application was also somewhat associated with suppressed H_2_O_2_ production, perhaps because melatonin can serve, with minimal toxicity, as a direct scavenger of free radicals and through a scavenging cascade [37,38]. In our experiments, MV stress induced greater production of MDA but melatonin significantly suppressed that response in pretreated leaves. This indicated that, although MV could cause oxidative stress by generating active oxygen species, melatonin alleviated lipid peroxidation.

Antioxidant enzymes are effective quenchers of ROS and play an important role in adaptations and ultimate survival by plants during oxidative stress. We investigated the activities of SOD (scavenging superoxide radicals and generating H_2_O_2_), CAT (scavenging H_2_O_2_), POD (scavenging H_2_O_2_), and enzymes involved in the ascorbate–glutathione (AsA–GSH) cycle (i.e., APX, MDHAR, DHAR, and GR) [28]. In higher plants, that cycle is a valuable antioxidant protection system against H_2_O_2_ generated in different cell compartments. Our results showed that the activities of ROS scavengers changed significantly and that different enzymes functioned at different times during the MV experiments. For example, SOD was primarily active at 12 and 24 h to scavenge superoxide radicals, while APX had a role in the first half of the oxidative stress period and CAT worked during the second half to scavenge H_2_O_2_. In particular, POD activity was significantly elevated between Hours 12 and 48. Similar patterns have been reported from a study of *Malus hupehensis* and exogenous melatonin, which relieved salinity-related oxidative damage by enhancing the activities of antioxidative APX, CAT, and POD [16]. In cucumber, pretreatment with melatonin led to greater activity by SOD, APX, and DHAR at 12 h after MV stress was induced while CAT activity remained largely unchanged [26]. Here, we found that all of the enzymes had roles in ROS-scavenging, albeit at different times during the period of MV treatment.

In this study, H_2_O_2_ (Figure 4A), MDA (Figure 4B), SOD (Figure 5A), CAT (Figure 5B), POD (Figure 5C), APX (Figure 5D) and the expression of related genes (Figure 6 and Figure 7) are mostly unaffected by added melatonin over the 48 h light exposure. After melatonin pretreatment, melatonin may improve capacity for stress tolerance, however, this capacity is not shown significantly before MV treatment. The protective role of melatonin is shown significantly after MV treatment. Our study highlights the melatonin action at the time of stress brought on by MV.

Pretreatment with melatonin also induced higher expression of *POD* at 48 h after exposure to MV. A similar pattern of expression was found with *CAT*. This demonstrated that enhanced expression later in the oxidative stress period is associated with a greater need for the protective effects of melatonin. Likewise, elevated expression of *AtAPX1* and *AtCAT*s improved the capacity to scavenge H_2_O_2_ in *Arabidopsis* seedlings under oxidative stress [25]. In our study, expression of *cAPX*, *cGR*, *MDHAR*, and *DHAR* increased mainly during the second half of the stress period, possibly helping to maintain a high level of AsA in pretreated leaves to fight against the effects of MV. This led us to deduce that melatonin first acts as a direct free radical scavenger and broad-spectrum antioxidant. It then increases the activity of antioxidant enzymes by enhancing related gene expression and cellular antioxidants such as ASA and GSH to protect apple leaves from oxidative damage [16,37,38].

Melatonin is synthesized from tryptophan sequentially by four enzymes. Genes for those enzymes were first identified in “Red Fuji” apple [39]. Expression of *MdTDC1*, *MdT5H4*, *MdAANAT2*, and *MdASMT1* was significantly upregulated by our MV treatment, which then caused additional melatonin to be produced in those plants. Melatonin pretreatment suppressed the expression of those genes during the subsequent period of oxidative stress. Similarly, in two *Malus* species tested under drought conditions, genes for melatonin synthesis were significantly upregulated by drought but that expression was suppressed in response to melatonin pretreatment [31]. This might have been accomplished due to high levels of endogenously produced melatonin in apple leaves and the inhibited activity of enzymes for melatonin synthesis that blocked the expression of related genes. Future investigations should focus on the role of redox signaling and the metabolites in melatonin that enable this molecule to regulate stress tolerance in plants.

## 4. Materials and Methods 

### 4.1. Plant Material and Treatments

Experiments were conducted at the College of Horticulture, Northwest A & F University, Yangling, China (34°20′ N, 108°24′ E). Fully mature leaves with complete petioles were detached from 2-year-old trees of “Fuji” apple (*Malus domestica* Borkh). They were wrapped with wet absorbent gauze for timely transport to the laboratory. After the leaves were cleaned with double-distilled water, the petioles were cut underwater to prevent gas bolt. The leaves were wrapped with cotton and transferred to plastic pots (47 cm × 35 cm × 10 cm) containing 0, 0.01, 0.1, 1, or 10 mM melatonin. Each dose group in this pretreatment contained three replicates of 40 leaves each. After 12 h of precultivation in the dark, each replicate was randomly divided into two portions. For one half, 500 μM MV was sprayed with a spray bottle on both sides of the leaves. For the other half (control), the leaves were sprayed with only double-distilled water. All of the leaves in these treatment groups were then transferred to a lighted chamber (100 μM photons m^−2^ s^−1^). After 48 h, the severity of damage for each leaf was scored on a scale of 0 to 5, where 0 = no damage symptoms, 1 = 1 to 10%, 2 = 11 to 30%, 3 = 31 to 50%, 4 = 51 to 70%, and 5 ≥ 70% of the leaf area showing lesions [18]. The following formula for disease index was used:

Damage index = (Sum of (Damage class × Number of leaves in that class) × 100)/(Total number of leaves × 5).

Based on preliminary results, we selected 1 mM melatonin for further study with 240 uniformly sized leaves. Half were pre-treated with 1 mM melatonin while the others received only double-distilled water. After 12 h of precultivation in the dark, the replicates were randomly assigned to two groups. For one half, 500 μM MV was sprayed with a spray bottle on both sides of the leaves while those in the other half (control) received only double-distilled water. All of the leaves were transferred to a lighted chamber (100 μM photons m^−2^ s^−1^). At Hours 0, 6, 12, 24, and 48 of MV exposure, leaf samples were collected, then quickly frozen in liquid nitrogen and stored at −80 °C.

### 4.2. Calculations of F_v_/F_m_, Chlorophyll Levels, and Electrolyte Leakage

Chlorophyll (Chl) fluorescence was measured with an integrating fluorescence fluorometer (LI-6400-40 Leaf Chamber Fluorometer; LICOR, Huntington Beach, CA, USA). After the samples were dark-adapted for 1 h, minimum fluorescence (*F_0_*) was measured under weak modulated irradiation (<0.1 μmol m^−2^ s^−1^). A 600-ms saturating flash (>7000 μmol m^−2^ s^−1^) was applied to determine the maximum Chl fluorescence yield (*F*_m_). Afterward, *F*_v_/*F*_m_ was calculated as (*F*_m_ *− F*_0_)/*F*_m_.

Chlorophyll was extracted with 80% acetone and concentrations were determined spectrophotometrically according to the method of Lichtenthaler and Wellburn [40]. Relative electrolyte leakage was determined according to the method described by Dionisio-Sese et al. [41]. 

### 4.3. Measurements of H_2_O_2_ and MDA

The H_2_O_2_ was extracted from leaf samples with 5% (*w*/*v*) trichloroacetic acid and measured as described by Patterson et al. [42]. After the amount of MDA was measured, the extent of lipid peroxidation in the leaves was assessed using 2-thiobarbituric acid as described by Hodges et al. [43]. 

### 4.4. Extraction and Assays of Antioxidant Enzymes

Leaf samples (0.1 g) were ground in a chilled mortar with 1% (*w*/*v*) polyvinylpolypyrrolidone, then homogenized with 1.2 mL of 50 mM potassium phosphate buffer (pH 7.8) containing 1 mM EDTA-Na_2_ and 0.3% Triton X-100. For the APX assay, 1 mM ascorbate was also added to this mixture. Each homogenate was centrifuged at 13,000× *g* for 20 min at 4 °C and the supernatant was used for the following assays.

Activity of CAT was determined by monitoring the decrease in absorbance at 240 nm due to decomposition of H_2_O_2_ (extinction coefficient of 39.4 mM^−1^ cm^−1^) [44], while that of APX was determined by monitoring the decrease in absorbance at 290 nm as reduced ASC was oxidized (extinction coefficient of 2.8 mM^−1^ cm^−1^) [45]. The activity of POD was assayed by monitoring the increase in absorbance at 470 nm due to guaiacol oxidation (extinction coefficient of 26.8 mM^−1^ cm^−1^) [46]. Finally, SOD activity was assayed by monitoring the inhibition of the photochemical reduction of nitro blue tetrazolium, according to the methods of Giannopolitis and Ries [47].

### 4.5. RNA Isolation and Quantitative Real-Time RT-PCR

Expression of *PAO*, genes coding for antioxidant enzymes, and those involved in AsA–GSH recycling or melatonin synthesis was evaluated with real-time quantitative reverse transcription–polymerase chain reactions (qRT-PCR). All primers used for qRT-PCR are given in Table 1.

Total RNA was extracted from the leaves according to the method described by Li et al. [16]. Poly(A)^+^ RNA was purified with a poly(A)^+^ Ttract^®^ mRNA Isolation Systems III kit (Promega, Madison, WI, USA) according to the manufacturer’s instructions. To remove any contaminating genomic DNA prior to cDNA synthesis, we treated the RNA with RNase-free DNase I (Invitrogen, Carlsbad, CA, USA) according to the manufacturer’s instructions. Total RNA was quantified on a NanoDrop™ 2000 spectrophotometer (Thermo Fisher, New York, NY, USA) before and after DNase I treatment, and its quality and integrity were checked by electrophoresis through agarose gels stained with ethidium bromide. All qRT-PCR procedures were performed using PrimeScript^TM^RT Reagent kits (Takara, Kyoto, Japan) with oligo (dT) 20 and random primers for cDNA synthesis, according to the manufacturer’s protocol. The amplified PCR products were quantified with an iQ5 Multicolor Real-Time PCR Detection System (Bio-Rad Laboratories, Hercules, CA, USA) and a SYBR Premix Ex Taq kit (Takara). Transcripts of the *Malus* elongation factor 1 alpha gene (*EF-1α*; DQ341381) were used to standardize the cDNA samples for different genes. These qRT-PCR experiments were repeated three times, based on three separate RNA extracts from three samples.

### 4.6. Statistical Analysis

The data were analyzed via one-way ANOVA, followed by Tukey multiple range tests. A *p*-value of <0.05 indicated a significant difference between treatments, and most data were presented as means ± standard deviation (SD) of three replicate samples. The exception was for Chl fluorescence measurements, which involved six replicates.

## 5. Conclusions

The application of melatonin can protect apple leaves from methyl viologen-mediated oxidative stress in a dose-dependent manner. Exogenous melatonin slows the decline in chlorophyll concentrations normally associated with MV exposure while also mitigating membrane damage, reducing lipid peroxidation, improving the activity of antioxidative enzymes. Genes for melatonin synthesis were significantly upregulated under oxidative stress in leaves but that expression was suppressed in response to melatonin pretreatment during the MV treatments. Future research will explore the underlying mechanisms for the relationships described here.

## Figures and Tables

**Table 1:** Sequences for primers used in quantitative real-time RT-PCR.

Gene	Primer Sequence (5′–3′)
*PAO*	F: ACCCGAGTGGTTTGGTACTTGTGA
	R: TACACGAGGAGCATTTGAGGGTGT
*CAT*	F: TGAAACCAAATCCAAAGACCA
	R: TTCCATGTGCCTGTAGTTGAGTG
*POD*	F: CCAACAAATGTGTCCCAAAAATG
	R: CCTGGTCCGAGGTAAATAATCC
*cAPX*	F: AACTACAAGGGATGAAGCC
	R: CAACGAGGATGATAACCAG
*cGR*	F: GTTCAGCGACAAGGCGTAT
	R: TCAACCGATTTCCATTTCC
*MDAR*	F: CCATACTTCTATTCCCGCTCCT
	R: CGACCACCTTCCCGTCTTT
*DHAR*	F: AGTGGACGGTTCCAGCAGA
	R: TTCCCATCCCGCAATCAC
*MdTDC1*	F: TCACGCTGTGGTTGGAGGT
	R: CTGCATGCTCCTGAACCAAC
*MdT5H4*	F: TCGGTGACATGTTTGCTGC
	R: GGAAACCTTGGTCTGGCG
*MdAANAT2*	F: GAATCACCGTCCACGCTCC
	R: GAAATGCTTCCGATGTCCC
*MdASMT1*	F: AGAGGAGCGAGAAAGACTGGA
	R: CTAAAGAAAAACTTCAATGAGGGAT
*EF-1α*	F: ATTCAAGTATGCCTGGGTGC
	R: CAGTCAGCCTGTGATGTTCC
